# Reassessing the Link between Airborne Arsenic Exposure among Anaconda Copper Smelter Workers and Multiple Causes of Death Using the Parametric g-Formula

**DOI:** 10.1289/EHP438

**Published:** 2016-08-19

**Authors:** Alexander P. Keil, David B. Richardson

**Affiliations:** Department of Epidemiology, University of North Carolina, Chapel Hill, Chapel Hill, North Carolina, USA

## Abstract

**Background:**

Prior studies have indicated associations between ingestion of inorganic arsenic and ischemic heart disease, nonmalignant respiratory disease, and lung, skin, bladder, and kidney cancers. In contrast, inhaled arsenic has been consistently associated only with lung cancer. Evidence for health effects of inhaled arsenic derives mainly from occupational studies that are subject to unique biases that may attenuate or obscure such associations.

**Objectives:**

We estimated the excess mortality from respiratory cancers, heart disease, and other causes resulting from occupational arsenic exposure while controlling for confounding using the parametric g-formula.

**Methods:**

Using a cohort of 8,014 male copper smelter workers who were hired between 1938 and 1955 and followed through 1990, we estimated the impacts of hypothetical workplace interventions on arsenic exposure on the risk of mortality from all causes, heart disease, and lung cancer using the parametric g-formula.

**Results:**

We estimate that eliminating arsenic exposure at work would have prevented 22 deaths by age 70 per 1,000 workers [95% confidence interval (CI): 10, 35]. Of those 22 excess deaths, we estimate that 7.2 (95% CI: –1.2, 15) would be due to heart disease, 4.0 (95% CI: –0.8, 8.2) due to respiratory cancers, and 11 (95% CI: 0.0, 23) due to other causes.

**Conclusions:**

Our analyses suggest that the excess deaths from causes other than respiratory cancers comprise the majority of the excess deaths caused by inhaled arsenic exposure. Healthy worker survivor bias may have masked such associations in previous analyses. These results emphasize the need for consideration of all exposure routes for upcoming risk assessment by the U.S. Environmental Protection Agency.

**Citation:**

Keil AP, Richardson DB. 2017. Reassessing the link between airborne arsenic exposure among Anaconda copper smelter workers and multiple causes of death using the parametric g-formula. Environ Health Perspect 125:608–614; http://dx.doi.org/10.1289/EHP438

## Introduction

Inorganic arsenic is a naturally occurring set of compounds that is classified as a known human carcinogen and is of potential concern because of its ubiquity in soil and water and the systemic nature by which it influences disease in humans (IARC et al. 2004; U.S. EPA 2001). Evidence regarding the association between ingested arsenic and human health has come mainly from populations exposed to high levels of arsenic in drinking water. In those studies, arsenic has been associated with increases in all-cause mortality [e.g., Argos et al. (2010)], ischemic heart disease [e.g., Tseng et al. (2003)], nonmalignant respiratory disease [e.g., Mazumder et al. (2000)], and cancers at sites including lung, skin, bladder, and kidney [e.g., Wu et al. (1989)]. In contrast, most epidemiological evidence regarding the health effects of inhaled inorganic arsenic comes from studies of exposure in workplaces. In such settings, the most consistent evidence of association with exposure to arsenic has been for lung cancer [e.g., Lubin et al. (2000)]. There is disagreement about whether data on airborne arsenic exposure provide evidence of associations with other health outcomes [e.g., Hertz-Picciotto et al. (2000); Lubin and Fraumeni (2000)]. Lung cancer is relatively rare. Thus, the population impact of airborne arsenic exposure has been described mainly by high relative rates for a rare disease, which limits the apparent magnitude of the potential public health impact.

It is not clear whether the lack of associations between airborne arsenic exposure and other causes of death is due to differences between the health effects by exposure route or whether it is due to bias that obscures health effect estimates in occupational studies. Previous authors have speculated that such associations may be masked by healthy worker survivor bias (Arrighi and Hertz-Picciotto 1994), a bias in occupational studies that may obscure causal relationships. Healthy worker survivor bias occurs when healthier workers (those experiencing low rates of disease) are able to sustain employment longer than sicker workers, thus accruing greater exposure over time. Some authors assert that healthy worker survivor bias does not appreciably affect estimates of the health effects of arsenic (Lubin and Fraumeni 2000). We propose that progress in assessing the magnitude of the public health impact of inhaled arsenic can be made by reanalyzing occupational data using methods that can improve control of healthy worker survivor bias. Under the assumptions that will be discussed in this article, such methods can improve the accuracy of estimates of the public health impact of exposure.

Healthy worker survivor bias has been conceptualized as time-varying confounding by employment status (Hertz-Picciotto et al. 2000), which in some cases cannot be controlled in the multivariable regression models (Buckley et al. 2015) used in previous analyses. The parametric g-formula (hereafter, “g-formula”) can be used to appropriately control confounding in such settings (Robins 1986). We have used the g-formula and data from a cohort of arsenic-exposed copper smelters to estimate the effects of hypothetical interventions on occupational arsenic exposure on mortality from heart disease, respiratory cancers, and all other causes combined. We report the results from a range of hypothetical interventions to illustrate expected changes in mortality with increasing arsenic exposure.

## Methods

### Study Population

Our study population is a cohort of workers from a copper smelting facility in Anaconda, Montana, followed from 1938 through 1990. This cohort, referred to as the Lee–Fraumeni cohort, has been described in detail elsewhere (Lee and Fraumeni 1969). Briefly, the cohort comprises 8,014 white, male individuals who worked ≥ 1 year at the smelter between 1 January 1938 and 31 December 1956. Follow-up began after the worker was employed for 1 year.

Employment records provided the work area and dates of job changes or termination. Records spanned the start of employment through 30 September 1977, when 11% of the workforce remained. From 1977 to 1980, when the smelter ceased operations permanently (Mercier 2001), no employment history is available.

Exposure to arsenic was quantified using work-area measurements from a series of 702 measurements of airborne arsenic trioxide (As_2_O_3_) obtained between 1943 and 1958 (Lee and Fraumeni 1969). From these measurements, personal exposure was classified as years employed in “heavy,” “medium” or “light” exposed jobs. These classifications were then used to create a quantitative exposure variable. The measurements were used to estimate a time-weighted airborne concentration for each work area corresponding to 0.29 mg/m^3^ (light), 0.58 mg/m^3^ (medium), and 11.4 mg/m^3^ (heavy). Following the procedure described by Lubin et al. (2008), we created a quantitative exposure metric in milligrams/cubic meter-years as the product of the duration of work and the airborne concentration in each area: *d* = 0.29 × years-light + 0.58 × years-medium + γ × 11.4 × years-heavy, where *d* is estimated exposure, γ is a weight, and years-light/medium/heavy are years employed in each job. The exposure metric was down-weighted for working in “heavy” exposed areas using γ = 0.1 to reflect the use of filtration masks (Lubin et al. 2000).

Using social security numbers provided by the company, the original investigators determined mortality from state health departments, Social Security claims records, insurance records, and the National Death Index. Causes of death were classified according to the *International Classification of Disease*, revision 8a (ICD-8a) as assigned to the underlying cause of death noted on death certificates [all deaths were coded to ICD-8a using an ICD cross-walk that was supplied to us by the lead author of Lubin et al. (2008)]. We created separate indicator variables for deaths caused by respiratory cancer (ICD-8a codes 160–163), heart disease [ICD-8a codes 410–414, 420–429; previously analyzed as “cardiovascular disease” by Hertz-Picciotto et al. (2000)], or all other causes (including deaths with unknown causes). The ICD codes that we used to define each outcome (see Table S1) were selected to maximize comparability to previous research in this cohort. Following the methods used by previous authors (Lubin et al. 2000, 2008; Robins 1986), we considered all individuals alive and at-risk through the earliest of the date of death, age 90, or the end of follow-up on 31 December 1990.

Age was the time scale for our analysis. We were primarily interested in the worker cohort as our target of inference. Thus, our analysis focused on contrasting the survival experience of the cohort under different interventions that could have been performed at the copper smelter to decrease (or increase) arsenic exposure.

### Statistical Methods

We estimated the cumulative incidence, or risk, from age 20 onwards, for three causes of death. The cumulative incidence of death at age *a* is the probability that a person has died due to an outcome of interest by the time they have reached age *a* (Cole et al. 2015). In a closed cohort, cumulative incidence corresponds to the observed proportion of deaths from a specific cause. In contrast with other common survival analysis estimands for cause-specific mortality, such as the Kaplan–Meier estimator (Kaplan and Meier 1958), the cumulative incidence does not depend upon the hypothetical removal of competing risks (Prentice et al. 1978).

The g-formula (Robins 1986) can be used to estimate the cumulative incidence using a time-varying version of standardization. Such an approach is not subject to bias when confounders may be affected by the exposure of interest. In occupational studies, employment status is hypothesized to be one such confounder (Buckley et al. 2015). In regression approaches, bias can result either through adjusting away part of the exposure or by conditioning on a variable that results in the creation of noncausal association, referred to as collider bias. In the g-formula, the estimated cumulative incidence is standardized (rather than stratified) over levels of confounders, some of which may be affected by exposure. Thus, the g-formula can be used to control confounding while avoiding bias from stratifying on a variable affected by exposure. We used the g-formula to estimate the cumulative incidence of death from heart disease, respiratory cancer, and all other causes. When follow-up occurs over a long period of time or continuous covariates are used, the g-formula can be approximated using parametric models and a Monte Carlo algorithm (Keil et al. 2014). This approach involves fitting separate models for each outcome of interest, for each time-varying confounder, for loss to follow-up (when it occurs), and for exposure (in some cases, discussed below). The Monte Carlo algorithm involves sampling repeatedly from the data and simulating the values of confounders and outcomes predicted from the parametric models. Using these predicted values, we can estimate the expected cumulative incidence under a set of interventions on our exposure of interest.

We focused our analysis on comparing mortality in the cohort under workplace interventions that could have been implemented in 1938, when follow-up started. Approximately one-third of the workers were hired before 1938. We consider exposure before 1938 as a potential confounder of the association of interest: mortality and arsenic exposure that occurred after enrollment. Thus, we are comparing the effects of interventions that could have been implemented in 1938.

Complete elimination of exposure at work would likely be infeasible given the available technology, but it is nonetheless informative with respect to estimating the excess burden of disease from occupational exposure. Therefore, we compared the cumulative incidence under different exposure scenarios with the cumulative incidence that would have been observed had exposure been eliminated for all person-time under follow-up (referred to as the “always unexposed” intervention). Following Robins (1986), our “always unexposed” intervention required an assumption that the effects of arsenic follow the parametric form specified in the models because the cohort included no unexposed workers. This assumption is commonly made in the arsenic literature, in which a linear model is frequently adopted (Lubin et al. 2008). We contrasted the cumulative incidence under the intervention “always unexposed” with the cumulative incidence that would have been observed under the “natural course,” which represented the action of no change in exposure but improved study design to ensure complete follow-up. The natural course recreated the observed history of exposure, confounders, and causes of death using the models described above plus a model for exposure while at work. Use of the natural course as a comparator (instead of the observed data) helped to isolate differences in mortality among hypothetical interventions that were due solely to exposure rather than to the combined influence of exposure and incomplete follow-up. Even under limited loss to follow-up, use of the natural course yielded cumulative incidence estimates with lower variance relative to estimates from the observed data. In addition, we contrasted the cumulative incidence for each outcome under three interventions that would have resulted in “heavy,” “medium,” or “light” exposure for all employees while at work. These comparisons allowed an informal analysis of exposure–response and illustrated the mortality trends that might have occurred had changes to industrial hygiene practices (to reduce occupational exposure) been implemented.

To estimate the parameters of the g-formula, we created parametric models for the following features of the data: death from respiratory cancer, death from heart disease, other causes of death, leaving work, returning to work, and exposure at work (light, medium or heavy). Individuals were considered to be at risk up until they were confirmed to be dead or were censored at age 90 years or the end of follow-up, so no model for loss to follow-up was needed. For death and employment variables, we fit pooled logistic models. For exposure, we fit a pooled, ordinal logistic [proportional odds (McCullagh 1980)] model for categories of exposure (light, medium, heavy). For each model, we chose a set of candidate model forms based on the Akaike Information Criterion (AIC). The final set of models was chosen according to how well the joint model predictions matched the observed data, based on comparisons of the mortality rates and average exposures between the natural course and the observed data. All models included baseline covariates [location of birth (United States or other), time worked before 1938 (linear term), occupational arsenic exposure before 1938 (linear, using the quantitative value described above)] and time-varying covariates [active work status (yes or no), cumulative time at work after 1938 (linear), cumulative arsenic exposure after 1938 (quantitative value, lagged 2 years allowing for different exposure coefficients by time since exposure (2–5 years, 5–10 years, 10–20 years) and assuming no associations with exposure more than 20 years prior]. Cause-specific mortality model fit, based on the AIC, worsened after including exposure more than 20 years prior, so it was kept out of the models. We modeled age-specific intercepts using a restricted cubic spline and adjusted for calendar time (linear) and age by time interaction terms to allow for birth cohort and/or period differences in baseline rates of the outcomes. Specific model forms are listed in Tables S2 and S3. The discrete-time rates of disease and employment changes under each intervention were simulated under each intervention using a Monte Carlo algorithm we have described previously (Keil et al. 2014), which numerically approximates the rates using simulated outcomes. Using the simulated outcomes, we estimated the cause-specific risk from age 20 with an extension of the Kaplan–Meier estimator that allows for competing risks to account for late entry on the age time-scale (Taubman et al. 2009). Accounting for late entry was necessary because not all workers were under observation at age 20.

We estimated the risk difference by subtracting the cumulative incidence in the natural course intervention from the always unexposed intervention. We focused on the estimation and precision of our results rather than on hypothesis testing (Wasserstein and Lazar 2016). We report the statistical precision of our results using bootstrap, percentile-based 95% confidence intervals (CIs) for the risk difference using a nonparametric, bootstrap standard error (1,500 iterations). The cumulative incidence was used to estimate the number of deaths per 1,000 workers for each cause (cumulative incidence × 1,000), and the risk difference was used to estimate the number of excess deaths per 1,000 resulting from exposure (risk difference × 1,000). We focused our analysis primarily on mortality at age 70. To assess differences in excess mortality by age we also report on mortality at age 60.

All analyses were performed using standard procedures in SAS 9.4 (SAS Institute Inc.) following the methods described in a previous analysis (Keil et al. 2014). Original data collection procedures have been described in detail by Lee and Fraumeni (1969). Deidentified data for the current manuscript were provided to the authors from the National Cancer Institute. This study was approved by the University of North Carolina Institutional Review Board committee, which granted a waiver of informed consent.

## Results

**Figure 1:**
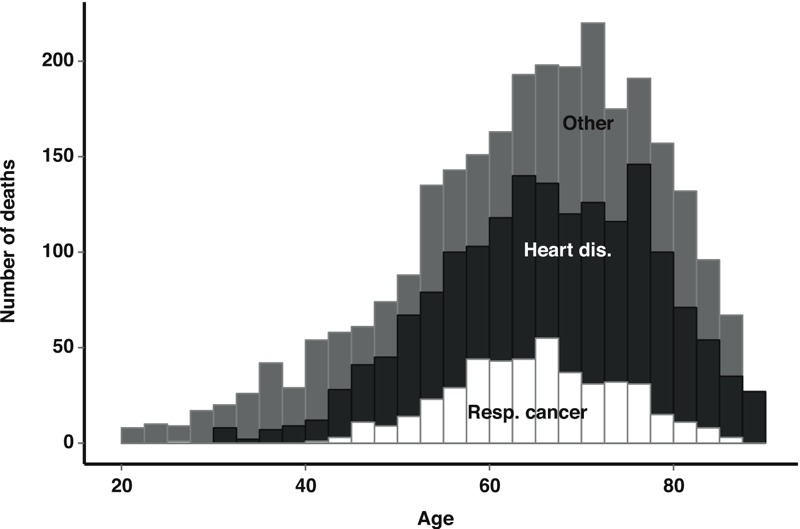
Age at death distribution for respiratory cancer (Resp. cancer), heart disease (Heart dis.), and other causes. The study population comprised 8,014 copper smelter workers, Anaconda, Montana, 1938–1990.

The median age at entry was 32, and the median time of work prior to study entry was 1 year (Table 1). Most workers were born in the United States. By the end of follow-up in 1990, only 39% were still alive, with 21% having died with heart disease as the underlying cause and 6% having died with respiratory cancer as the underlying cause. As shown in Figure 1, the distributions of observed ages at death were similar among respiratory cancer [median, interquartile range (IQR) = 65, 58–71], heart disease (median, IQR = 67, 58–75) and deaths from other causes (median, IQR = 66, 56–75).

**Table 1:** Demographic, exposure, and vital status characteristics of the study population, 8,014 copper smelter workers, Anaconda, Montana, 1938–1990.

Characteristic	Median (IQR)^*a*^
Age at study entry	31.6 (23.2–43.6)
Age at hire	25.3 (19.6–35.3)
Age at last employment	46.4 (32.2–60.6)
Date of birth (year)	1912 (1901–1922)
Date of hire	1942 (1929–1949)
Arsenic exposure at entry
Exposed, number (%)	7,802 (97.4)
Cumulative mg/m^3^-years	0.29 (0.23–0.77)
Years employed at entry	0.99 (0.81–1.83)
U.S. born, number (%)	6,945 (86.7)
Vital status, number (%)^*b*^
Alive	3,136 (39.1)
Deceased, respiratory cancer^*c*^	445 (5.6)
Deceased, heart disease^*d*^	1,690 (21.1)
Deceased, other or unknown cause	2,743 (34.2)
IQR, interquartile range. ^***a***^Median (IQR) unless otherwise indicated. ^***b***^Vital status as of the earlier of age 90 or 31 December 1990. ^***c***^*International Classification of Diseases*, revision 8a, codes 160–164. ^***d***^*International Classification of Diseases*, revision 8a, codes 410–414, 420–429.	

**Figure 2:**
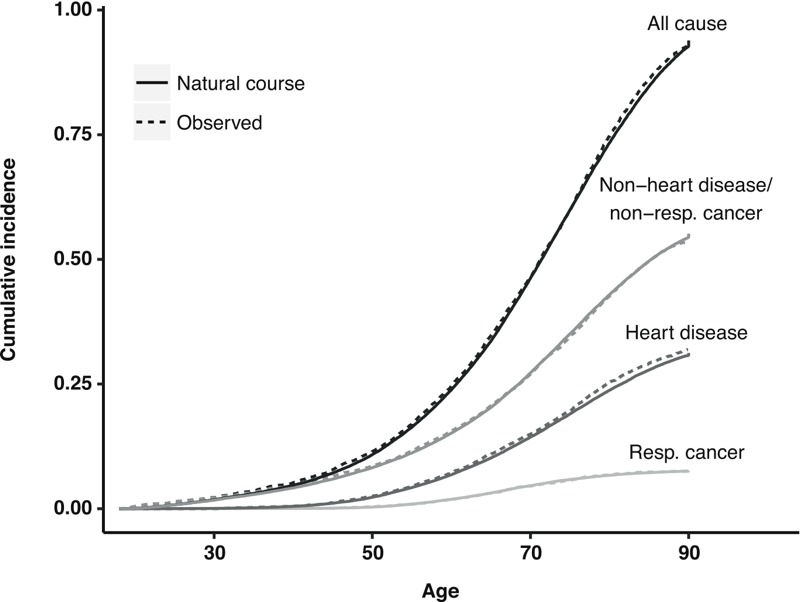
Cumulative incidence estimates for the observed data (dashed lines) and under the “natural course” intervention in the g-formula (solid lines) for all-cause and cause-specific mortality [respiratory (Resp.) cancer mortality (*International Classification of Diseases*, revision 8a codes 160–164); heart disease mortality (cardiovascular disease; *International Classification of Diseases*, revision 8a codes 410–414, 420–429)]. The study population comprised 8,014 copper smelter workers, Anaconda, Montana, 1938–1990.

The cumulative incidence functions for respiratory cancer, heart disease, and other causes showed good correspondence between the observed data and the cumulative incidence predicted for the natural course (Figure 2). The observed median cumulative arsenic exposure across all person-time in the study was 1.7 mg/m^3^-years (interquartile range: 0.87–4.1). The predicted exposure under the natural course intervention corresponded well with the observed data [median, IQR = 1.7 (0.87–4.4) mg/m^3^-years]. A 1-mg/m^3^-year increase in exposure during the preceding 1–5 years increased the log-odds of leaving work by 2.3 (standard error = 0.02), suggesting that healthy worker survivor bias in this cohort could not be controlled by simply adjusting for employment status in a regression model because it is associated with prior exposure.

**Figure 3:**
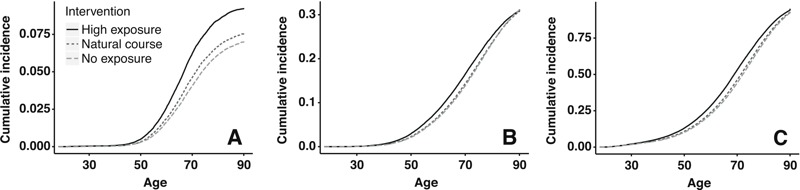
Cumulative incidence curve under hypothetical interventions on arsenic exposure for respiratory cancer mortality (*A*), heart disease mortality (*B*) and all-cause mortality (*C*). The study population comprised 8,014 copper smelter workers, Anaconda, Montana, 1938–1990. Light/medium exposure interventions not included for clarity.

The cumulative incidence of respiratory cancer by age 70 (46 deaths per 1,000) was approximately one-third that of heart disease (150 deaths per 1,000) and one-fifth that for other causes of death (270 per 1,000). We estimated that arsenic exposure resulted in an excess of 22 (95% CI: 10, 35) deaths from all causes per 1,000 individuals (the sum of all cause-specific excess deaths), of which we attribute 7.2 (95% CI: –1.1, 15) to heart disease, 4.0 (95% CI: –0.8, 8.2) to respiratory cancer, and the remaining 11 (95% CI: 0.0, 15) to other causes (Table 2). Under the intervention “if at work, receive heavy exposure,” we estimate that all specific causes of death would have been elevated relative to the natural course. Excess mortality under the interventions “if at work receive light/moderate exposure” were intermediate between the “always unexposed” and the “heavy exposure” interventions. By age 90, the risk difference between the natural course and the “never exposed” intervention had diminished for cardiovascular disease and all-cause mortality, whereas it had grown for lung cancer mortality (Figure 3). The estimate of excess deaths was generally lower and more precise for each cause of death at age 60 versus age 70 (Table 2).

**Table 2:** Cause-specific and all-cause mortality per 1,000 and excess deaths per 1,000 at age 60 and age 70.

Age (years)/Cause of mortality	Deaths per 1,000^*a*^ (95% CI)	Excess deaths per 1,000^*b*^ (95% CI)			
	No exposure	Natural course	If at work, light exposure	If at work, medium exposure	If at work, heavy exposure
Age 60
All causes	224 (211, 239)	14 (5.0, 22.3)	12 (4.1, 20)	27 (14, 40)	60 (33, 88)
Respiratory cancer	17 (13, 20.2)	1.7 (–0.4, 3.9)	1.6 (–0.5, 3.7)	4.0 (0.6, 7.3)	10 (2.6, 20)
Heart disease	65 (58, 73)	4.8 (0.2, 9.1)	4.1 (–0.4, 8.4)	8.7 (1.4, 16)	18 (2.8, 34)
Other causes	143 (132, 156)	7.3 (–0.1, 15)	6.5 (–0.3, 14)	14 (2.3, 26)	32 (8.0, 58)
Age 70
All causes	441 (423, 460)	22 (10, 35)	20 (8.3, 31)	42 (23, 62)	89 (51, 128)
Respiratory cancer	42 (35, 50)	4.0 (–0.8, 8.2)	3.6 (–0.7, 7.4)	8.9 (0.7, 16)	21 (2.3, 43)
Heart disease	138 (126, 152)	7.2 (–1.1, 15)	6.4 (–1.2, 13)	13 (–0.9, 26)	25 (–2.5, 54)
Other causes	261 (244, 279)	11 (0.0, 23)	9.9 (–0.7, 21)	20 (1.8, 40)	43 (4.2, 83)
CI, confidence interval. The cohort comprised 8,014 copper smelter workers, Anaconda, Montana, 1938–1990. ^***a***^Cumulative incidence × 1,000. ^***b***^Risk difference × 1,000 (relative to no exposure; negative values imply that higher exposures would decrease the risk of mortality).					

## Discussion

We estimated that an intervention in 1938 to eliminate arsenic exposure in this cohort of copper smelters would have delayed approximately 22 deaths per 1,000 by age 70. The estimated effect of reducing exposure relative to the natural course, measured by the excess deaths due to exposure at age 70, is nearly as high among causes other than respiratory cancer and heart disease mortality as it is among the excess deaths for those two causes combined. Estimated excess deaths from other causes were not influenced greatly by other malignant causes of concern, such as bladder and skin cancer, which comprised < 5% of the 1,010 cancer deaths (data not shown). Our results are consistent with the hypothesis that airborne arsenic acts systemically to influence nonmalignant and nonrespiratory fatal diseases. As in a prior report on the association between arsenic and circulatory diseases in this cohort (Lubin and Fraumeni 2000), the estimated cumulative incidence differences for heart disease mortality were not statistically significant at age 70. However, our findings suggest that the public health burden of mortality (at age 70) for heart disease and respiratory cancer may be similar, if not higher for heart disease. The estimated impact from other causes of death was higher still.

Authors of previous reports on the same cohort of copper smelters have concluded that airborne arsenic exposure is not associated with an increase in heart disease mortality (Lubin and Fraumeni 2000; Lubin et al. 2000). Our results are not formally comparable to previous research on this topic. Lubin et al. (2000) reported standardized mortality ratios, which rely on a referent group from the general population. The proportion of smokers in a subset of this cohort was estimated to be ~80%, which makes internal comparisons desirable for smoking-related diseases such as respiratory cancer and heart disease (Welch et al. 1982). Our results are a comparison of the same group of workers under different hypothetical scenarios, which increases comparability in mortality risk factors, such as smoking, among exposure groups. Lubin and Fraumeni (2000) reported stratified rate ratios, which are also not directly comparable with our results because we report marginal effect estimates on a different scale and over a different age range. Thus, we could only make qualitative comparisons to the prior literature. In contrast to Lubin and Fraumeni’s interpretation of their results, we inferred that arsenic exposure was associated with an increase in the risk of both respiratory cancer and heart disease at age 70. After adjusting for baseline mortality differences between the workers and the general population, Lubin et al. (2008) estimated standardized mortality ratios that were elevated but not statistically significant (α = 0.1) at cumulative exposure levels < 10 mg/m^3^-years. Similarly, we estimated that the respiratory cancer excess estimate was low, relative to its precision, for the lowest exposures.

Our estimates of excess lung cancer are consistent with prior analyses of these data (Lubin et al. 2008), and perhaps coherent with inhalation as the primary exposure route in this occupational setting. However, arsenic ingestion through drinking water is also associated with lung cancer (Celik et al. 2008), and a pooled dose–response analysis by Smith et al. suggested that the association between urinary arsenic and lung cancer is similar, regardless of whether arsenic exposure is from drinking water or smelter work (Smith et al. 2009). Thus, the exposure route may not be the driving force between the lack of association between arsenic and heart disease in previous occupational studies. Our recent work has shown that healthy worker survivor bias in occupational studies may result in potentially large underestimates of exposure–response metrics for mortality, including lung cancer (Keil et al. 2015), and the association between prior exposure and employment status suggests that such bias may have occurred in previous analyses of this cohort. Healthy worker survivor bias may explain, in part, why occupational studies often do not estimate positive associations between arsenic exposure and heart disease.

Much of the evidence for the health effects of chronic arsenic exposure comes from observational studies, which are primarily restricted to the ingestion exposure route. Further, we found no experimental studies that focused on cardiovascular outcomes associated with inhalation of arsenic trioxide. Little is known about how inorganic arsenic absorption differs between ingestion and inhalation routes, although arsenic trioxide is readily soluble in the lungs (WHO 2001). Arsenic-containing dusts may be ingested as well as inhaled (Roels et al. 1982), or they may be transported from the lungs to the mouth via mucociliary clearance. Thus, we would expect that occupational studies would replicate many of the findings of the large population-based studies of arsenic ingestion.

The choice of a relevant age is key to interpreting our results. We chose to focus on the mortality risk at age 70 because at this age, individuals would be expected to maintain a high quality of life, but employment-based exposures would likely have already begun to show any potential effects. Our results comparing the mortality risk among hypothetical interventions depend on this *a priori* choice both qualitatively and statistically. The number of excess deaths from exposure depends strongly on age, as shown in Table 2 and Figure 3, and statistical precision will generally be lower at older ages. The results at age 60 were generally more precise than those at age 70 (Table 2). We could have alternatively quantified mortality using estimands such as the average lifespan. This measure is not straightforward to interpret when considering specific causes of death, however, because exposure may accelerate the disease course or cause death from a different disease (Morfeld 2004; Robins and Greenland 1991). Our data were not sufficient to differentiate between these two potential mechanisms, and excess risk provides a summary measure of the total impact of exposure.

According to our analysis, the association between arsenic and heart disease was stronger in intermediate age ranges (Figure 3). These results are consistent with previous analyses of this cohort in which early analysis indicated an excess of heart diseases relative to the general population (Lee and Fraumeni 1969; Lubin et al. 1981; Welch et al. 1982), whereas analysis after many more years of follow-up did not (Lubin et al. 2000). As an alternative measure of arsenic’s impact across the life course, the g-formula allowed us to calculate years of life lost for all causes by simply comparing the person-time under each intervention, which suggested an overall detriment that is not apparent in the cumulative incidence at age 90. This approach is not possible for specific causes of death, however (Morfeld 2004; Robins and Greenland 1991). Because heart diseases may reduce the quality of life for many years before death, the true public health burden from airborne arsenic exposure may lie mainly in its effects on cardiovascular outcomes. This is a limitation of using mortality as an end point, which is likely a better indication of incident disease for respiratory cancer (which is characterized by low survival) than for heart disease.

We focused on mortality from respiratory cancer (85% of which was lung cancer deaths) and heart disease primarily for comparability to prior analyses, where disagreement regarding healthy worker survivor bias and the health effects of airborne arsenic centered primarily on these two outcomes (Arrighi and Hertz-Picciotto 1996; Hertz-Picciotto et al. 2000; Lubin and Fraumeni 2000; Lubin et al. 2008). In an analysis of another smelter cohort, Hertz-Picciotto et al. reported a positive exposure–response between arsenic and heart disease (termed “cardiovascular disease” by those authors) after attempting to reduce healthy worker survivor bias by adjusting for employment status (Hertz-Picciotto et al. 2000). In response to that article, Lubin analyzed data from the present cohort and suggested that there was no association between arsenic and heart disease, even after adjusting for employment status (Lubin and Fraumeni 2000). However, adjusting for employment status may not remove this bias (Buckley et al. 2015) and could potentially increase it (Keil et al. 2015). Both Hertz-Picciotto et al. (2000) and Lubin and Fraumeni (2000) focused on relative measures of effect, which can understate the apparent public health impact of common diseases. We elaborated on previous work by appropriately controlling for healthy worker survivor bias and by estimating absolute effects of exposure using the g-formula. By estimating the effects of interventions that could decrease (“never exposed,” “if at work, receive low exposure”) as well as increase (“if at work, receive medium/heavy exposure”) exposure relative to the natural course, we informally assessed the exposure–response curve.

Healthy worker survivor bias can arise when employment status acts as a confounder of the association of interest (Buckley et al. 2015). Employment status is consistently associated with many health outcomes and is a strong (often deterministic) predictor of subsequent exposure. In our study, employment status was independently (from other confounders) associated with prior exposure, thus fitting the criteria for a time-varying confounder affected by prior exposure. Confounding by such variables can be controlled by the g-formula but not by multivariable regression (Keil et al. 2014). Under the assumptions of noninterference (one person’s exposure cannot affect another’s outcome), correct model specification, positivity (intervention levels of exposure are possible within all strata of confounders), and no unmeasured confounding (conditional exchangeability), the g-formula can be used to estimate the distributions of health outcomes that we would expect under interventions on exposure, such as changes in regulatory conditions or workplace policies (Robins 1986). Although our approach included exposure–response trends through parametric modeling, our primary results are expressed as expected excess mortality under a discrete set of interventions. Based on these results, we expect larger excesses of death resulting from respiratory cancer, heart disease, and other causes at higher exposures.

Compared with standard regression analyses, the parametric g-formula is more sensitive to the assumption of correct model specification, an assumption that will never hold exactly (Robins 1986). However, we assert that our models held at least approximately because we favored model flexibility over model parsimony. The results shown in Figure 1 support this claim because for the natural-course intervention, our models closely matched what was observed in the data. Although the agreement between the observed survival times and the predictions from standard regression models can be used to assess model fit, comparisons are not often made between model predictions and observed data, which represents an advantage to our approach. A further advantage of the g-formula over standard regression models is that even with highly flexible models that allow for nonlinearity and interactions, the inference remains a simple contrast of mortality under a limited number of hypothetical scenarios, as in a clinical trial. As is the case in all approaches that rely on modeling, however, there is no guarantee that models are correctly specified, and bias may result. If, for example, our model for heart disease underestimated the rate at zero exposure but not at higher levels of exposure, then we would likely overestimate the effect of arsenic on heart disease. Such an error could occur, for example, if the true exposure response followed a threshold model. With respect to lung cancer, the results reported by Lubin et al. (2008) suggest that linear extrapolation of the excess rate gives a reasonable approximation of the exposure–response curve at low exposures.

As occurs with many occupational cohort studies, we lack information on potential confounders of associations between occupational exposures and mortality, such as smoking. We did not have smoking data and were thus unable to directly control for smoking, which is a cause of both lung cancer and heart disease. Previous authors have observed that workers in the copper smelter cohort with persistent work in high-exposure jobs had a 2–5% larger proportion of smokers than those with persistent work in lower-exposure jobs (Welch et al. 1982). However, this small difference is unlikely to explain our results. Our approach assumed that, conditional on the modeled covariates, annual arsenic exposure is not associated with smoking. Such an association could arise if, for example, smokers were preferentially placed into higher- (or lower-) exposed jobs. However, if smoking affects exposure only by its effects on one’s ability to stay employed (or if employment and smoking status share a common cause), we could control unmeasured confounding by annual smoking simply by adjusting for employment status. Thus, smoking could be one reason why adjusting for employment status is important to control healthy worker survivor bias (Buckley et al. 2015). Other measures or determinants of health status, such as undiagnosed incident cardiovascular disease, were also unmeasured and may play a similar role in healthy worker survivor bias.

Ideally, we would like to estimate the impact of interventions on all workers who worked at the Anaconda smelter, which was built in 1919. However, all cohort members had to have worked ≥ 1 year at the smelter after 1938 before becoming eligible for the cohort. The long-term workers who were working in 1938 (33% of the cohort) likely had different prognoses from those of former workers of a similar age and date of hire because those with poor prognosis would likely have already left employment or died and consequently have become ineligible for the study, a process referred to as left truncation (Applebaum et al. 2011). Thus, we could estimate the effects of hypothetical interventions before 1938, and our estimates of excess risk were conditional on being alive and employed through the beginning of entry into the study. Because we could estimate the excess mortality at different exposure levels, our results directly estimated the population impact of exposure and the possible effects that implementing different exposure standards may have had in this cohort. The generalizability of our estimates to other populations is uncertain given that the workers were all employed males and not representative of the U.S. population. Similarly, predicting risk into the future for current workers under changing regulations is difficult because of secular trends in disease and demographic differences between worker populations. Specific conditions under which our results could be formally generalized or transported to a broader population are discussed by Pearl and Bareinboim (2014).

Measurement error, in both the exposure and causes of death, is an important limitation to our study. Our quantitative estimate of arsenic exposure is based on work-area means. The exposure assessment approach is liable to both classical and Berkson-type measurement errors, which may bias cumulative exposure–response associations and may also substantially reduce the precision of estimated associations (Armstrong 1998). In the g-formula, prior exposure is treated as a confounder, so measurement error in the exposure may also lead to residual confounding. Further, this study relied on cause-of-death information from death certificates; errors resulting from imperfect sensitivity and specificity of the death certificate for classification of the underlying cause of death may be additional sources of bias and imprecision in these estimates. The accuracy of coding for cardiovascular events, such as heart disease, may have changed over the course of follow-up as diagnostic procedures improved. The extensive modeling in the parametric g-formula may increase sensitivity to measurement error, but the magnitude of these biases is unknown and is an area of active research.

## Conclusion

Our findings have implications for highly exposed occupational groups, such as smelters, among whom healthy worker survivor bias may result in underestimation of the detrimental effects of arsenic. Ambient arsenic exposure is generally low compared with ingestion (European Commission 2001), but these findings suggest a need for further research regarding the cardiovascular effects of airborne arsenic exposure in certain nonoccupational settings where airborne exposure may be nonnegligible, such as those burning coal within the home (Liu et al. 2002). As of Summer 2016, arsenic has been under consideration for risk assessment by the U.S. Environmental Protection Agency, and the public health impacts of exposure from all sources should be considered.

## Supplemental Material

(120 KB) PDF
