# Supplementary material for: Reassessing the Link between Airborne Arsenic Exposure among Anaconda Copper Smelter Workers and Multiple Causes of Death Using the Parametric g-Formula
Source: Environ Health Perspect. 2016 Aug 19;125(4):608–14. doi: 10.1289/EHP438 (PMC5381993; doi:10.1289/EHP438)
Supplement: (120 KB) PDF [file EHP438.s001.acco.pdf]

Environ Health Perspect
DOI: 10.1289/EHP438

Note to readers with disabilities: *EHP* strives to ensure that all journal content is accessible to all readers. However, some figures and Supplemental Material published in *EHP* articles may not conform to 508 standards due to the complexity of the information being presented. If you need assistance accessing journal content, please contact ehp508@niehs.nih.gov. Our staff will work with you to assess and meet your accessibility needs within 3 working days.

Supplemental Material

Reassessing the Link between Airborne Arsenic Exposure among Anaconda Copper Smelter Workers and Multiple Causes of Death Using the Parametric g-Formula

Alexander P. Keil and David B. Richardson

Table of Contents

Table S1: ICD-8 codes used for specific causes of death
Table S2: Model forms
Table S3: Variable names and definitions

Table S1: ICD-8 codes used for specific causes of death

ICD-8 code	Description
	Respiratory cancer
160	Malignant neoplasm of nose, nasal cavities, middle ear and accessory sinuses
161	Malignant neoplasm of larynx
162	Malignant neoplasm of trachea, bronchus and lung
163	Malignant neoplasm of other and unspecified respiratory organs
	Heart disease
410-414	*Ischemic Heart disease*
410	Acute myocardial infarction
411	Other acute and subacute forms of ischaemic heart disease
412	Chronic ischaemic heart disease
413	Angina pectoris
414	Asymptomatic ischaemic heart disease
420-429	*Other forms of heart disease*
420	Acute pericarditis, non-rheumatic
421	Acute and subacute endocarditis
422	Acute myocarditis
423	Chronic disease of pericardium, non-rheumatic
424	Chronic disease of endocardium
425	Cardiomyopathy
426	Pulmonary heart disease
427	Symptomatic heart disease
428	Other myocardial insufficiency
429	Ill-defined heart disease

Table S2: Model forms

Regressand	Model form	Regressors[a]
Respiratory cancer mortality (binary)	Pooled logistic	cum_as_2_5, cum_as_5_10, cum_as_10_20, agein_cen, agein_cen1, agein_cen2, agein_cen3, caltime, agein_cen*caltime, agein_cen1*caltime, agein_cen2*caltime, agein_cen3*caltime, caltime*caltime, cumtawbfu, cumtawbfu*cumtawbfu, usborn, cum_as_score_bfu, inactivelag1, activework, cumtawdfu_lag1, cumtawdfu_lag1*cumtawdfu_lag1
Heart disease mortality (binary)	Pooled logistic	cum_as_2_5, cum_as_5_10, cum_as_10_20, agein_cen, agein_cen1, agein_cen2, agein_cen3, caltime, agein_cen*caltime, agein_cen1*caltime, agein_cen2*caltime, agein_cen3*caltime, cumtawbfu, cumtawbfu*cumtawbfu, usborn, cum_as_score_bfu, inactivelag1, activework, cumtawdfu_lag1, cumtawdfu_lag1*cumtawdfu_lag1
All other causes of death (binary)	Pooled logistic	cum_as_2_5, cum_as_5_10, cum_as_10_20, agein_cen, agein_cen1, agein_cen2, agein_cen3, caltime, agein_cen*caltime, agein_cen1*caltime, agein_cen2*caltime, agein_cen3*caltime, cumtawbfu, cumtawbfu*cumtawbfu, usborn, cum_as_score_bfu, inactivelag1, activework, cumtawdfu_lag1, cumtawdfu_lag1*cumtawdfu_lag1
Leaving work, pre-1977 (binary)	Pooled logistic	cum_as_2_5, cum_as_5_10, cum_as_10_20,agein_cen_aw*over65, agen_cen_aw*(1-over65), cumtawbfu, cumtawbfu*cumtawbfu, usborn, cum_as_score_bfu, cumtawdfu_lag1, cumtawdfu, cumtawdfu_lag1*cumtawdfu_lag1
Returning to work, pre-1977 (binary)	Pooled logistic	cum_as_1_5, cum_as_5_10, cum_as_10_20, agein_cen_ow, agein_cen_ow1, agein_cen_ow2, agein_cen_ow3, caltime_ow, agein_cen_ow*caltime_ow, agein_cen_ow1*caltime_ow, agein_cen_ow2*caltime_ow, agein_cen_ow3*caltime_ow, cumtawbfu, cumtawbfu*cumtawbfu, usborn, cum_as_score_bfu, over65, cumtawdfu_lag1, cumtawdfu_lag1*cumtawdfu_lag1
Arsenic exposure at work (Low, medium, heavy exposure)	Pooled cumulative logistic	cum_as_1_5, cum_as_5_10, cum_as_10_20, agein_cen, agein_cen1, agein_cen2, agein_cen3, caltime, agein_cen*caltime, agein_cen1*caltime, agein_cen2*caltime, agein_cen3*caltime, cumtawbfu, cumtawbfu*cumtawbfu, usborn, cum_as_score_bfu, cumtawdfu_lag1, cumtawdfu_lag1*cumtawdfu_lag1

[a] Variable names defined in Table S3.

Table S3: Variable names and definitions

Variable name	Description
activework	Currently at work during person period
agein_cen	Age at start of person-period, centered
agein_cen1	Restricted cubic spline variable 1, age at start of person period
agein_cen1_aw	Restricted cubic spline variable 1, age at start of person period, knots defined using percentiles of person time of actively employed workers
agein_cen1_ow	Restricted cubic spline variable 1, age at start of person period, knots defined using percentiles of person time of inactive workers
agein_cen2	Restricted cubic spline variable 2, age at start of person period
agein_cen2_aw	Restricted cubic spline variable 2, age at start of person period, knots defined using percentiles of person time of actively employed workers
agein_cen2_ow	Restricted cubic spline variable 2, age at start of person period, knots defined using percentiles of person time of inactive workers
agein_cen3	Restricted cubic spline variable 3, age at start of person period
agein_cen3_aw	Restricted cubic spline variable 3, age at start of person period, knots defined using percentiles of person time of actively employed workers
agein_cen3_ow	Restricted cubic spline variable 3, age at start of person period, knots defined using percentiles of person time of inactive workers
caltime	Calendar time at start of person period (centered at 1/1/1950, per 20 years)
cum_as_1_5	Total arsenic exposure from 1-5 years prior to start of person period
cum_as_10_20	Total arsenic exposure from 10-20 years prior to start of person period (quantitative score)
cum_as_2_5	Total arsenic exposure from 2-5 years prior to start of person period (quantitative score)
cum_as_5_10	Total arsenic exposure from 5-10 years prior to start of person period (quantitative score)
cum_as_score_bfu	Total arsenic exposure from before follow-up (quantitative score)
cumtawbfu	Cumulative time at work before follow-up (years)
cumtawdfu	Cumulative time at work during follow-up (years)
cumtawdfu_lag1	Cumulative time at work during follow-up (lag 1 year)
inactivelag1	Off work for more than one year (lag 1 year, 1=yes, 0=no)
over65	Age 65 or over (1:age>65, 0:age<=65)
usborn	Born in the United states (1=yes, 0=no)
